# Suprapedicular Circumferential Opening Technique of Percutaneous Endoscopic Transforaminal Lumbar Discectomy for High Grade Inferiorly Migrated Lumbar Disc Herniation

**DOI:** 10.1155/2018/5349680

**Published:** 2018-01-18

**Authors:** Hyeun Sung Kim, Farid Yudoyono, Byapak Paudel, Ki Joon Kim, Jee-Soo Jang, Jeong-Hoon Choi, Sung Kyun Chung, Jeong Hoon Kim, Il-Tae Jang, Seong-Hoon Oh, Jae Eun Park, Sol Lee

**Affiliations:** ^1^Department of Neurosurgery, Nanoori Suwon Hospital, Suwon, Republic of Korea; ^2^Department of Neurosurgery, Hasan Sadikin Hospital, College of Medicine, Padjadjaran University, Bandung, Jawa Barat, Indonesia; ^3^Department of Orthopaedics, Grande International Hospital, Kathmandu, Nepal; ^4^Department of Neurosurgery, Nanoori Hospital, Seoul, Republic of Korea; ^5^Department of Neurosurgery, Nanoori Incheon Hospital, Incheon, Republic of Korea; ^6^Nanoori Medical Research Institute, Nanoori Hospital, Seoul, Republic of Korea

## Abstract

**Purpose:**

To evaluate the efficacy of suprapedicular circumferential opening technique (SCOT) of percutaneous endoscopic transforaminal lumbar discectomy (PETLD) for high grade inferiorly migrated lumbar disc herniation.

**Material and Methods:**

Eighteen consecutive patients who presented with back and leg pain with a single-level high grade inferiorly migrated lumbar disc herniation were included. High grade inferiorly migrated disc was removed by the SCOT through PETLD approach. Outcome evaluation was done with visual analog scale (VAS) and Mac Nab's criteria.

**Result:**

There were 14 males and 4 females. The mean age of patients was 53.3 ± 14.12 years. One, 4, and 13 patients had disc herniation at L1-2, L3-4, and L4-5 levels, respectively, on MRI, which correlated with clinical findings. The mean follow-up duration was 8.4 ± 4.31 months. According to Mac Nab's criteria, 9 patients (50%) reported excellent and the remaining 9 patients (50%) reported good outcomes. The mean preoperative and postoperative VAS for leg pain were 7.36 ± 0.73 and 1.45 ± 0.60, respectively (*p* < 0.001). Improvement in outcomes was maintained even at final follow-up. There was no complication.

**Conclusion:**

In this preliminary study we achieved good to excellent clinical results using the SCOT of PETLD for high grade inferiorly migrated lumbar disc herniation.

## 1. Introduction

Microdiscectomy is known as a gold standard surgical procedure for lumbar disc herniation (LDH) [1–6]. However, percutaneous endoscopic discectomy of the LDH has progressed considerably since introduction of the concept by Kambin in the year 1973 [1, 7–11]. Recently, percutaneous endoscopic transforaminal lumbar discectomy (PETLD) has gained popularity to treat LDH, due to lesser injury inflicted to the posterior spinal muscle, lesser iatrogenic instability, smaller scar in the epidural space, and lesser retraction of the neural tissue. Because of the difficulty in accessing the migrated disc due to the presence of anatomical barriers like pedicle and narrowing of the foraminal space, PETLD has not been considered as an optimal treatment option for a high grade inferiorly migrated disc along the traversing root [9, 11–15]. High grade inferiorly migrated LDH is defined as a disc migration beyond the inferior margin of the pedicle.

Success rate of the surgery in the high grade inferiorly migrated LDH might be low due to remnants of the disc fragment [9, 13, 14]. Advances in surgical equipment present an opportunity to improve the surgical efficacy [5, 15, 16] for high grade inferiorly migrated LDH. We applied a transforaminal suprapedicular circumferential opening technique (SCOT) for a high grade inferiorly migrated LDH. We want to share our technique and results.

## 2. Materials and Methods

Eighteen consecutive patients treated between November 2015 and October 2016 using the SCOT were reviewed retrospectively. The inclusion criteria were as follows: (a) patients presented with back pain and leg pain with a single-level high grade inferiorly migrated lumbar disc herniation, confirmed by magnetic resonance imaging (MRI) and (b) failure of conservative therapies over 4–6 weeks. The exclusion criteria were definitive segmental instability, foraminal stenosis, and spondylotic spondylolisthesis. X-ray and CT scan were done routinely preoperatively in all patients in addition to MRI to aid in diagnosis and preoperative planning.

### 2.1. Surgical Technique

Patient was placed in prone position over Wilson frame on a radiolucent operation table. Under fluoroscopic guidance, entry point was marked. The entry point and trajectory of needle were infiltrated with 1% lidocaine. The spinal needle was inserted toward the lowest part and most dorsal part of disc space. The foraminal space is infiltrated with 7–10 cc 1% lidocaine followed by epinephrine mixed 2-3 cc 1.6% lidocaine, 3–5 minutes after the first injection [6, 7, 14, 17, 18]. At a midpoint between the end plates, discography was performed, using a contrast mixture consisting of 6 mL iohexol dye and 1 mL indigo carmine, which is a nontoxic marker for staining of the disc material and has no neurotoxicity. A guide-wire was inserted through the needle into the intervertebral disc. After withdrawing the needle, a tapered cannulated obturator was slipped over the guide-wire and advanced into the foraminal space. Working channel was passed over the obturator. After docking the bevel type working channel into the foraminal space, obturator was removed. An endoscope (Joimax GmbH, Raumfabrik 33A, Amalienbadstraße, Karlsruhe, Germany) was inserted through the working channel.

**Figure 1:**
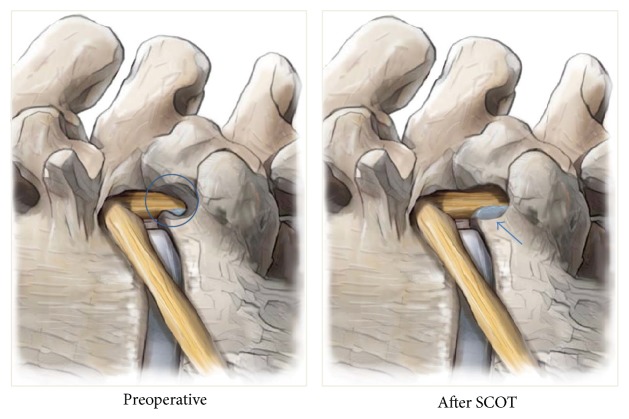
Illustration of SCOT procedure (lateral projection). Drilling area (blue circle) and visualization of high grade inferiorly migrated disc herniation after SCOT procedure (arrow).

**Figure 2:**
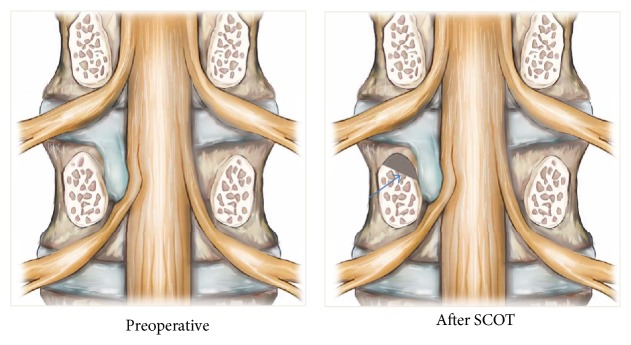
Illustration of SCOT procedure (coronal projection). Drilling area (brown colored) suprapedicular notch (arrow).

**Figure 3:**
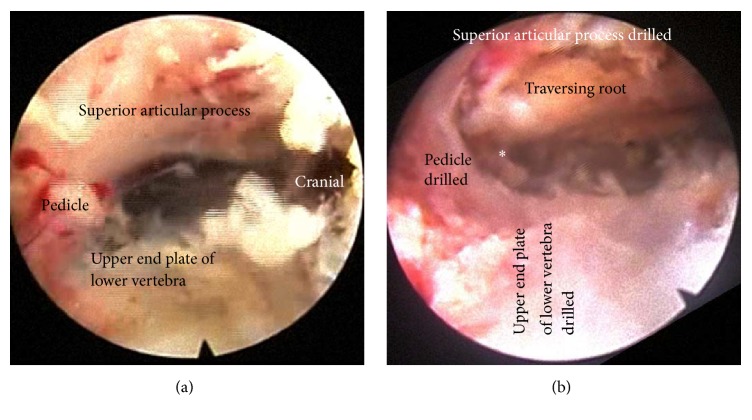
Endoscopic view. (a) Initial endoscopic view around the foramen of SCOT procedure. Suprapedicular notch, upper endplate of lower vertebrae, and Superior articular process, (b) after SCOT procedure by drilling (widening of neuroforamen seen). ^*∗*^Window to migrated disc.

**Figure 4:**
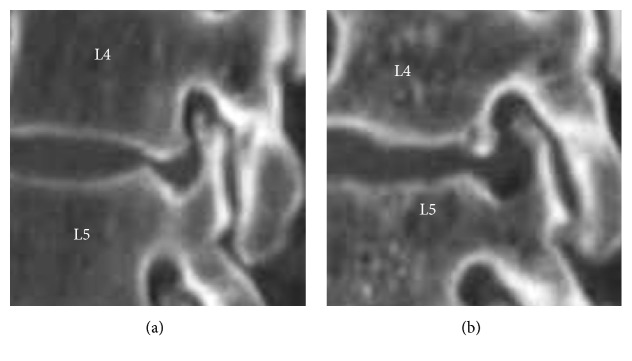
CT images of SCOT. (a) preoperative; (b) postoperative.

After completing internal disc decompression, the working channel was then directed to suprapedicular notch [16]. The pedicle is surrounded by ample soft tissue, which has to be removed completely using a radiofrequency coagulator (Elliquence, New York, USA) and forceps. SCOT is performed by drilling (Primado 2, NSK, Tochigi, Japan) (1) the ventral part of superior articular process, (2) the upper part of pedicle that builds the suprapedicular notch, and (3) the upper-posterior margin of the lower vertebra, to increase the width of the foramen and expose the ventral epidural space. During the procedure, the blue-stained disc fragment was visible below the traversing nerve root after the SCOT. (Figures 1, 2, 3, and 4)

If straight probe or forceps was unable to reach the inferior migrated herniated disc due to anatomic limitations, we carefully used semirigid flexible curved probe and forceps to hook and pull the disc material out. Then, a straight or curved forceps could reach the disc fragment and we could easily remove it. Bleeding was controlled with a radiofrequency probe and continuous normal saline irrigation. The traversing nerve root was then assessed. Finally, the working cannula and the endoscope were removed. MRI was taken 4~24 hours after SCOT to confirm removal of the migrated disc fragment.

The surgical outcomes were assessed using Mac Nab's criteria and the Visual Analog Score (VAS).

## 3. Results

A total of 18 patients were operated on during the period between November 2015 and October 2016, for high grade inferiorly migrated lumbar disc herniation using the SCOT through PETLD approach. The mean age of the patients was 53.3 ± 14.12 years; 14 patients were male and 4 were females. One, 4, and 13 patients had LDH at the L1-2, L3-4, and L4-5 levels, respectively, on MRI, which correlated with clinical findings. The mean pre- and postoperative VAS for leg pain was 7.36 ± 0.73 points and 1.45 ± 0.60 (*p* < 0.001) (Table 1).

The mean duration of follow-up was 8.41 ± 4.31 months. According to Mac Nab's criteria, 9 patients (50%) rated their well-being as excellent, and 9 patients (50%) rated it as good. The patients' VAS for leg pain and Mac Nab's criteria were maintained till the last follow-up from postoperative status.

There was no case of infection, discitis, paresis, dural tear, vascular injuries, or systemic complications until the last follow-up.

**Figure 5:**
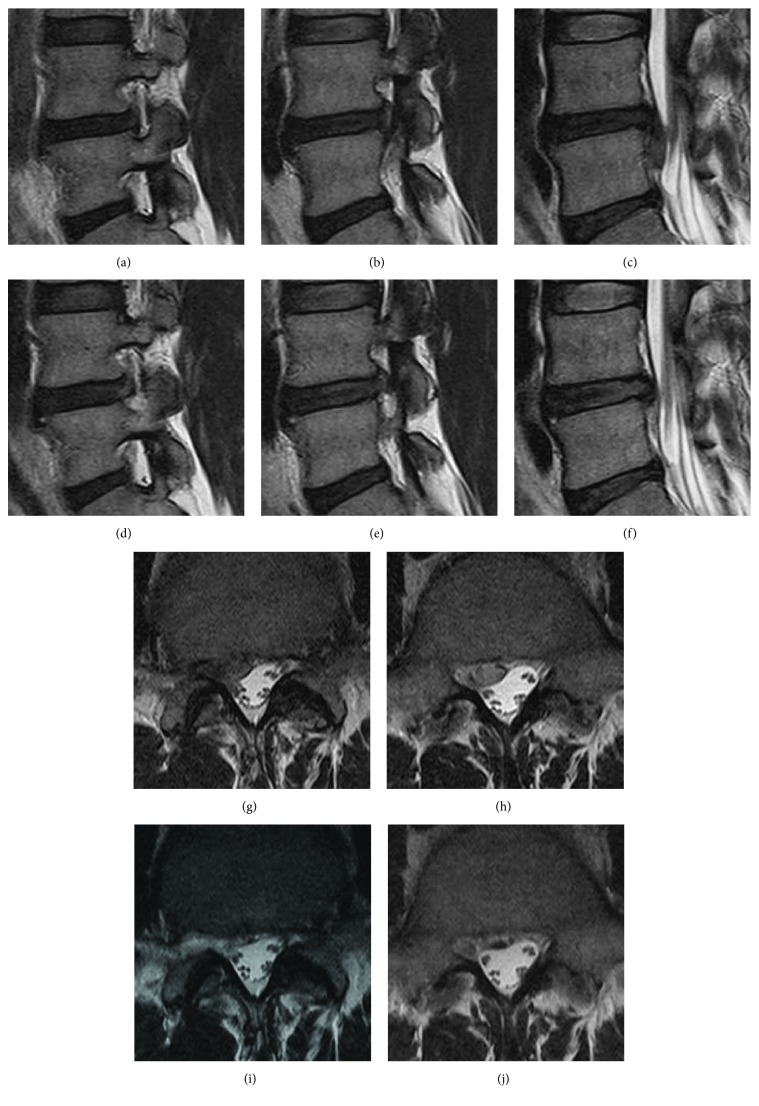
Representative case serial MRI of SCOT. (a), (b), (c), (g), (h) Preoperative MRI showing high grade inferior migration at L4-5 level. (d), (e), (f), (i), (j) Postoperative MRI showing complete removal of high grade inferiorly migrated herniated disc. (a), (d) Entry part of SCOT, (b), (e) middle part of SCOT, and (g), (h) final part of SCOT.

**Figure 6:**
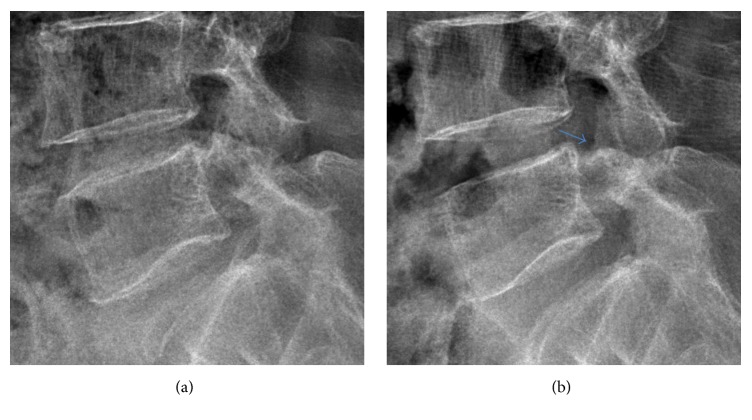
Radiologic illustration of SCOT in lumbar X-ray lateral view. (a) Preoperative X-ray. (b) Postoperative X-ray showing widened neuroforamen without structural damage (blue arrow).

Postoperative MRI revealed complete disc removal in all cases. Representative case is shown in Figure 5 (MRI). Postoperative X-ray did not reveal the any significant damage to any drilled structures. But, foramen was found widened. Representative X-ray is shown in Figure 6.

## 4. Discussion

Since Kambin introduced the posterolateral percutaneous nucleotomy technique in 1973, minimally invasive surgery has gained prominence in all fields of surgery. Recently, numerous studies involving endoscopic discectomy have been reported. However, PETLD is focused only on internal decompression for reduction of the intradiscal pressure. With advancement of surgical technology, different types of disc herniation are now accessible through endoscopy. Previous challenges such as high migration, far lateral fragment, and sequestrated discs are now being easily dealt with [19–21]. However, for high grade inferiorly migrated disc herniation, the procedure is technically demanding, with a less favorable outcome [13, 14, 16]. Revolutionary advent of surgical instruments and advanced endoscopic techniques [21] has enabled PETLD to provide good surgical outcomes [11, 13–15] for the treatment of high grade inferiorly migrated disc herniation. Improvements in technique and surgical equipment [5, 15, 16] have reduced the limitations for surgical management caused by the anatomic barrier. Lee et al. [8] reported that inadequate decompression and remnant disc result in endoscopic discectomy failure and increase the rate of repeated surgery. These differ significantly according to the size and location of the herniation [13].

In previous surgical approaches, like foraminoplastic out-and-in technique, the point of entry is slightly cranial to the facet joint and the guide rod is docked firmly between the lower vertebral body and superior articular process (SAP). The tip of the guide rod contacts the dorsal surface of lower vertebral body at the mid portion. Dorsal surface of the distal guide rod shaft contacts the ventral surface of SAP around the foramen. Part of it is removed with the reamer and the neuroforamen is widened. In TESSYS out-and-in technique, the point of entry is the intervertebral foramen, and superior margin of the lower vertebral body is removed to widen the neuroforamen. In suprapedicular technique, the entry point is upper margin of the lower vertebral pedicle. In occurrence of spondylosis, upper margin of lower vertebral is removed and neuroforamen is widened [16, 22, 23]. Previous techniques have limitations for high grade inferiorly migrated disc.

SCOT is a new technique for removal of high grade inferior migrated disc, through the narrow foraminal space above the pedicle of the lower vertebra. SCOT has several advantages; prone position provides easier accessibility and does not damage the native structures. It has lower probability of intradiscal damage, and the risk of injury to the dura mater is relatively low. It has an angle that allows access to the migrated disc herniation and enables sufficient assessment of traversing root decompression as well as disc extraction.

Epidural bleeding may occur during the SCOT from vascular engorgement. To avoid this, care should be taken when placing the endoscopic instrument in the epidural space. A careful approach along the migrated disc herniation to the tip of the fragment is essential. Hemostasis can be achieved with radiofrequency coagulator, high pressure fluid irrigation, or hemostatic agent such as absorbable gelatin sponges, if inevitable. In this study, no complication of dura tear occurred during surgery, as there is a clear boundary between the thecal sac and the migrated disc capsule [5, 7]. We had no conversion to the open surgery due to dural injury or surgical failure; however, some cases may need conversion to open surgery. Therefore, the surgeon should always confirm the anatomical relationship between the nerve and soft tissues, through the endoscopic visual field.

The limitations of this study are the relatively short follow-up period and small sample size. Considering the low incidence of high grade inferiorly migrated disc herniation in clinical practice, our number of cases may be considerable. We believe that a longer follow-up period with a larger number of cases is necessary to evaluate definitive effect of SCOT.

Despite the limitations, our results show that SCOT is a safe and reliable method to achieve a favorable clinical outcome in limited indications for PETLD of high grade inferiorly migrated lumbar disc herniation.

## 5. Conclusion

In this preliminary study, we achieved good to excellent clinical results using the SCOT of percutaneous transforaminal endoscopic discectomy for high grade inferiorly migrated lumbar disc herniation.

## Figures and Tables

**Table 1:** Pre- and postoperative VAS (leg).

		Preoperative		Postoperative		*p value*
		Mean	(±SD)	Mean	(±SD)	
VAS		7.36	±0.73	1.45	±0.60	*<0.001* ^**∗**^

^**∗**^ *p* < 0.001.

## References

[1] Kambin P., Savitz M. H. (2000). Arthroscopic microdiscectomy: an alternative to open disc surgery. *Mount Sinai Journal of Medicine*.

[2] Tzaan W.-C. (2007). Transforaminal percutaneous endoscopic lumbar discectomy. *Chang Gung Medical Journal*.

[3] Choi G., Modi H. N., Prada N. (2013). Clinical results of XMR-assisted percutaneous transforaminal endoscopic lumbar discectomy. *Journal of Orthopaedic Surgery and Research*.

[4] Gadjradj P. S., Van Tulder M. W., Dirven C. M., Peul W. C., Harhangi B. S. (2016). Clinical outcomes after percutaneous transforaminal endoscopic discectomy for lumbar disc herniation: a prospective case series. *Neurosurgical Focus*.

[5] Ahn Y. (2012). Transforaminal percutaneous endoscopic lumbar discectomy: Technical tips to prevent complications. *Expert Review of Medical Devices*.

[6] Mayer H. M., Brock M. (1993). Percutaneous endoscopic discectomy: surgical technique and preliminary results compared to microsurgical discectomy. *Journal of Neurosurgery*.

[7] Ahn Y., Jang I., Kim W. (2016). Transforaminal percutaneous endoscopic lumbar discectomy for very high-grade migrated disc herniation. *Clinical Neurology and Neurosurgery*.

[8] Lee S., Kang B., Ahn Y. (2006). Operative failure of percutaneous endoscopic lumbar discectomy: a radiologic analysis of 55 cases. *Spine*.

[9] Choi G., Lee S.-H., Raiturker P. P., Lee S., Chae Y.-S. (2006). Percutaneous endoscopic interlaminar discectomy for intracanalicular disc herniations at L5-S1 using a rigid working channel endoscope. *Neurosurgery*.

[10] Choi G., Lee S.-H., Bhanot A., Raiturker P. P., Chae Y. S. (2007). Percutaneous endoscopic discectomy for extraforaminal lumbar disc herniations: extraforaminal targeted fragmentectomy technique using working channel endoscope. *The Spine Journal*.

[11] Yeung A. T., Yeung C. A. (2003). Advances in endoscopic disc and spine surgery: foraminal approach.. *Surgical Technology International*.

[12] Yeung A. T., Tsou P. M. (2002). Posterolateral endoscopic excision for lumbar disc herniation: surgical technique, outcome, and complications in 307 consecutive cases. *The Spine Journal*.

[13] Schubert M., Hoogland T. (2005). Endoscopic transforaminal nucleotomy with foraminoplasty for lumbar disk herniation. *Operative Orthopädie und Traumatologie*.

[14] Lee S. H., Kang B. U., Ahn Y. (2006). Operative failure of percutaneous endoscopic lumbar discectomy: a radiologic analysis of 55 cases. *Spine (Phila Pa 1976)*.

[15] Kim C. H., Chung C. K., Woo J. W. (2012). Surgical outcome of percutaneous endoscopic interlaminar lumbar discectomy for highly migrated disc herniation. *Journal of Spinal Disorders & Techniques*.

[16] Kim H. S., Ju C. I., Kim S. W., Kim J. G. (2009). Endoscopic transforaminal suprapedicular approach in high grade inferior migrated lumbar disc herniation. *Journal of Korean Neurosurgical Society*.

[17] Choi G., Lee S.-H., Lokhande P. (2008). Percutaneous endoscopic approach for highly migrated intracanal disc herniations by foraminoplastic technique using rigid working channel endoscope. *The Spine Journal*.

[18] Tomecek F. J., Anthony C. S., Boxell C., Warren J. (2002). Discography interpretation and techniques in the lumbar spine.. *Neurosurgical Focus*.

[19] Lee S., Kim S.-K., Lee S.-H. (2007). Percutaneous endoscopic lumbar discectomy for migrated disc herniation: classification of disc migration and surgical approaches. *European Spine Journal*.

[20] Jang J.-S., An S.-H., Lee S.-H. (2006). Transforaminal percutaneous endoscopic discectomy in the treatment of foraminal and extraforaminal lumbar disc herniations. *Journal of Spinal Disorders & Techniques*.

[21] Tsou P. M., Yeung A. T. (2002). Transforaminal endoscopic decompression for radiculopathy secondary to intracanal noncontained lumbar disc herniations: outcome and technique. *The Spine Journal*.

[22] Lee C.-W., Yoon K.-J., Ha S.-S., Kang J.-K. (2016). Foraminoplastic superior vertebral notch approach with reamers in percutaneous endoscopic lumbar discectomy: technical note and clinical outcome in limited indications of percutaneous endoscopic lumbar discectomy. *Journal of Korean Neurosurgical Society*.

[23] Iprenburg M., Godschalx A. (2008). A Transforaminal Endoscopic Surgery in Lumbar Disc Herniation in an Economic Crisis—The TESSYS Method. *US Musculoskeletal Review*.

